# Multicenter and inter-software evaluation of ablative margins after thermal ablation of colorectal liver metastases

**DOI:** 10.1007/s00330-024-10956-5

**Published:** 2024-08-02

**Authors:** Gregor Laimer, Koen H. M. Verdonschot, Lina Kopf, Susan van der Lei, Yannick Scharll, Gerjon Hannink, Sjoerd F. M. Jenniskens, Martijn R. Meijerink, Reto Bale, Christiaan G. Overduin

**Affiliations:** 1 Department of Radiology, Interventional Oncology, Stereotaxy and Robotics, Medical University Innsbruck, Innsbruck, Austria; 2 Department of Medical Imaging, Radiology, Radboud University Medical Center, Nijmegen, Netherlands; 3 Department of Radiology and Nuclear Medicine, Amsterdam UMC location Vrije Universiteit Amsterdam, Amsterdam, Netherlands

**Keywords:** Liver neoplasms, Radiofrequency ablation, Imaging (three-dimensional), Treatment outcome

## Abstract

**Purpose:**

To assess the association between minimal ablative margin (MAM) and local tumor progression (LTP) following CT-guided thermal ablation of colorectal liver metastases (CRLM) in a multicenter cohort and across two confirmation software.

**Materials and methods:**

This multicenter retrospective study included patients who underwent CT-guided radiofrequency or microwave ablation for CRLM between 2009 and 2021 in three institutions. Three-dimensional (3D) MAM was retrospectively assessed using dedicated ablation confirmation software by automatic non-rigid (Ablation-fit) or semi-automatic rigid co-registration (SAFIR) of intraprocedural pre- and post-ablation contrast-enhanced CT scans by two independent reader teams blinded to patient outcomes. LTP was assessed on a per-tumor basis. Factors associated with LTP-free survival were assessed using multivariable Cox regression analysis.

**Results:**

Overall, 113 patients (mean age: 67 ± 10 years; 78 men) who underwent thermal ablation for 189 CRLM (mean diameter: 1.9 ± 1.1 cm) met the inclusion criteria. 173/189 (92%) CRLM could be successfully analyzed using both software. Over a median follow-up of 31 months (IQR: 22–47), 21 of 173 CRLM (12.1%) developed LTP. On multivariable analysis, 3D MAM was independently associated with LTP in both software (Ablation-fit: HR 0.47, 95% CI: 0.36–0.61, *p* < 0.001; SAFIR: HR 0.42, 95% CI: 0.32–0.55, *p* < 0.001). No LTP was observed in CRLM ablated with MAM ≥ 4 mm (Ablation-fit) and ≥ 5 mm (SAFIR). The per-tumor median absolute difference in MAM quantification between both software was 2 mm (IQR: 1–3).

**Conclusion:**

MAM was independently associated with LTP after thermal ablation of CRLM across multicenter data and two confirmation software. Ablations achieving a MAM ≥ 5 mm were associated with local control in both software.

**Clinical relevance statement:**

MAMs from intraprocedural contrast-enhanced CT were independently associated with LTP after thermal ablation of CRLM across multicenter data and two confirmation software, with a margin ≥ 5 mm associated with local control in both software.

**Key Points:**

- *Sufficient ablative margins are critical for local control following thermal ablation of CRLM*.
- *Intraprocedural CT-derived MAM was the only independent factor associated with LTP across two confirmation software.*
- *No LTP was observed in CRLM ablated with a MAM ≥* *5* *mm*.

## Introduction

Percutaneous thermal ablation is a well-established minimally invasive local treatment option for colorectal liver metastases (CRLM) with widely expanded indications over the past decade [1, 2]. Radiofrequency (RFA) and microwave ablation (MWA) are the most commonly used thermal ablative modalities and have been demonstrated as parenchyma-sparing treatment techniques with low rates of major complications and treatment-related mortality [3–5].

Despite these advantages, thermal ablation has historically been reserved for patients who are not eligible for surgery due to its relatively higher rates of local tumor progression (LTP) (2–60%) [6, 7]. Among several tumor- or patient-related characteristics, numerous studies have recently identified the minimal ablative margin (MAM) as the most critical predictor for LTP [8–11]. In a recent meta-analysis of retrospective studies, complete coverage of the target lesion with sufficient ablative margin (i.e., > 5–10 mm) was shown associated with reduced risk of LTP [12].

In current practice however, many physicians rely on assessing the MAM by a side-by-side comparison of pre- and post-interventional CT scans—a method shown to be poorly reproducible and unreliable for predicting treatment success in various studies [13, 14]. Image fusion by (semi-)automatic confirmation software with three-dimensional (3D) volumetric assessment has proven to overcome these difficulties [8, 15–18]. Currently available confirmation software solutions however have been reported mainly in retrospective, single-center studies with rather small sample sizes, providing an overall low level of evidence [19]. An extensive multi-institutional evaluation is presently lacking. Adding to this, the current literature is characterized by wide variation in margin quantification approaches [20] and the consistency of the optimal ablative margin across different software is still unknown.

The primary aim of this study was to assess the association between 3D MAM and LTP following CT-guided thermal ablation of CRLM in a multicenter cohort and across two confirmation software. The secondary objectives were to evaluate the diagnostic performance and inter-software consistency of MAM quantification using each software.

## Materials and methods

### Patients

This multicenter retrospective study included data from three academic institutions (Radboud University Medical Center [RUMC]; Medical University Innsbruck [MUI]; Amsterdam University Medical Center, location VUMC [VUMC]). The study was approved by the institutional review board at each institution and informed consent was waived. Institutional databases were queried to identify all patients that had undergone RFA or MWA for CRLM and met the following inclusion criteria: (i) treated for local treatment-naïve lesion(s); (ii) intraprocedural pre- and immediate post-ablation contrast-enhanced CT (CE-CT) imaging available; (iii) > 12 months imaging follow-up available. For RUMC, all consecutive patients treated between March 2015 and May 2021 were included. For MUI, all consecutive patients treated between January 2009 and October 2018 were included. For VUMC, a randomly selected subset of 55 patients treated between October 2014 and April 2020 was included. Part of the data (45 of 113 patients) used in this study overlaps with a previous study [16]. The prior study was a single-center study utilizing one confirmation software whereas in this study we report results in a multicenter cohort and all data was analyzed by a second confirmation software.

**Fig. 1:**
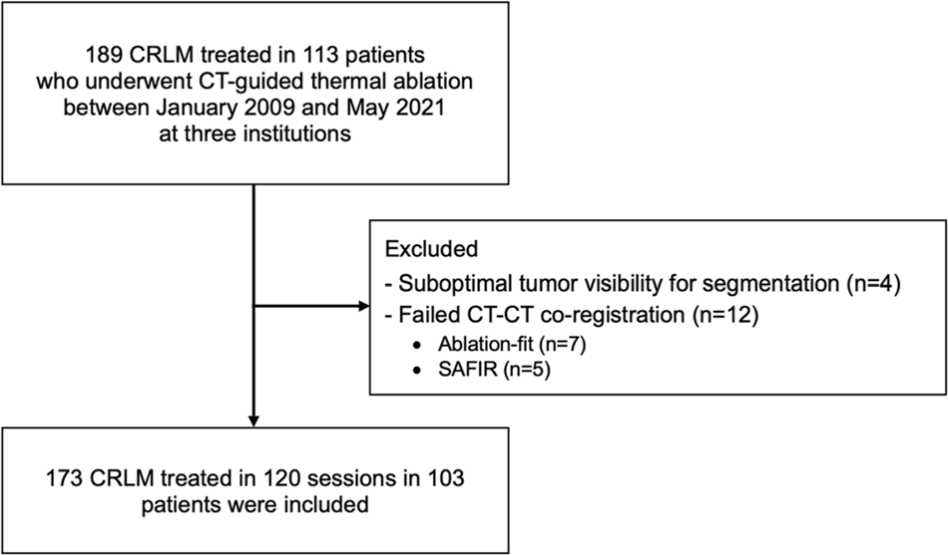
Flowchart of patient selection and criteria

In all patients, the decision to perform thermal ablation was made by the multidisciplinary tumor board. A detailed flowchart is shown in Fig. 1.

### Ablation procedures

All thermal ablation procedures were performed in the CT room by a board-certified interventional radiologist. Patients were treated under general anesthesia in two centers and under general anesthesia or propofol sedation in one center. Depending on local treatment protocol, intraprocedural pre- and immediate post-ablation CE-CT imaging was performed using either intravenous contrast injection or transcatheter hepatic arteriography (CTHA) [21]. In one center, a stereotactic navigation device was used in all cases for probe placement of which the methodology has been described in detail before [22, 23]. In the other two centers, probe placement was performed under ultrasound or CT fluoroscopy guidance. Thermal ablation was performed using RFA (Cool-tip, Covidien; LeVeen, Boston Scientific; Starburst, Angiodynamics) or MWA (Emprint, Medtronic; NeuWave, Ethicon; Solero, Angiodynamics) depending on site and historic availability. Technical success was defined as complete ablation with an ablative margin of ≥ 5 mm as evaluated at the end of each procedure by the treating physician on two-dimensional side-by-side comparison or rigid superposition of pre- and post-ablation CE-CT images.

### Outcome assessment

Ablation outcomes were assessed following standardized reporting guidelines for tumor ablation [3, 24]. All patients underwent routine imaging follow-up consisting of CE-CT, CE-MRI, or PET-CT imaging every 3 months for the first year followed by 3–6 month intervals thereafter. All follow-up imaging was reviewed by a board-certified abdominal radiologist. LTP was defined as tumor focus within or at the border of the ablation zone on follow-up imaging after the immediate post-ablation CE-CT documented complete ablation. Time to LTP was defined as the time between thermal ablation and imaging evidence of LTP.

### MAM quantification

All imaging data was independently reviewed at two institutions (RUMC and MUI). At each institution, a reader team consisting of a senior radiology resident and an interventional radiologist in consensus performed MAM quantification blinded to patient outcome using a dedicated ablation confirmation software: (i) Ablation-fit (R.A.W. SRL), commercially available software for liver thermal ablation, at MUI or (ii) Software Assistant for Interventional Radiology (SAFIR) (Fraunhofer MEVIS), research software for liver thermal ablation, at RUMC.

For both software, intraprocedural pre- and post-ablation arterial and portal-venous phase CE-CT scans (in-plane resolution 0.6–1.0 mm; slice thickness 0.5–3.0 mm) were imported. First, the liver parenchyma was automatically segmented and manually adjusted if needed. Second, in each software, each colorectal liver metastasis (CRLM) and corresponding coagulation zone were manually identified by the reader and semi-automatically segmented in the pre- and post-ablation CE-CT scans, respectively. Manual corrections were performed until segmentation results were deemed satisfactory. Third, the pre- and post-ablation CE-CT scans were co-registered. For Ablation-fit, an automatic non-rigid co-registration was performed by the software. For SAFIR, a semi-automatic rigid co-registration was performed and manually adjusted if needed. Co-registration was optimized based on local landmarks, in particular liver vessels, to obtain optimal local registration in the segment of interest. In both software, co-registration accuracy was assessed using a blending slider allowing to switch between pre- and post-ablation CE-CT scans. Insufficient co-registration accuracy was defined as a mismatch in more than one local landmark (e.g., vessels) of > 3 mm, and such cases were excluded from further MAM quantification.

**Fig. 2:**
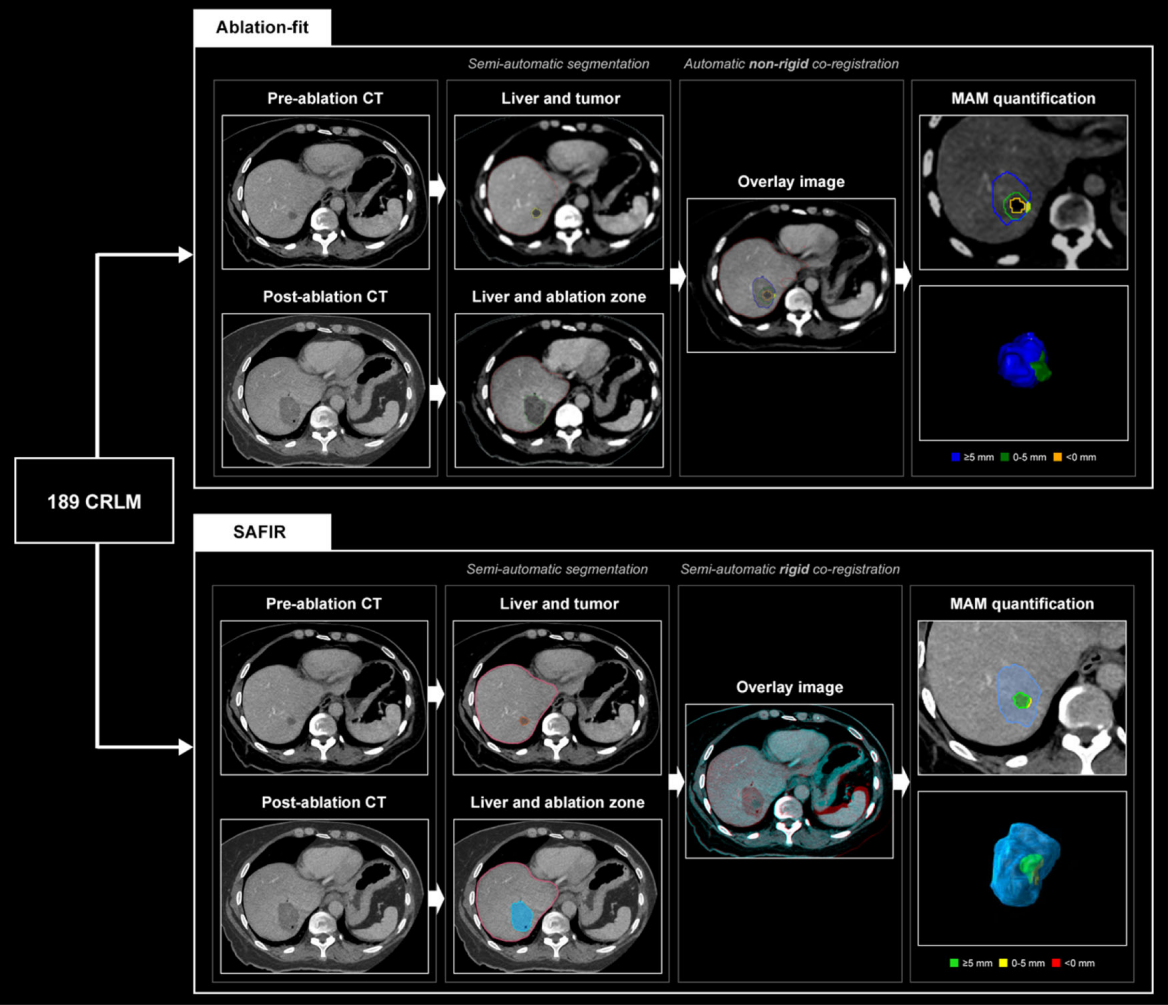
Schematic overview of MAM quantification steps for each software. Semi-automatic segmentation of the liver, tumor, and ablation zone is performed on the pre- and post-ablation CE-CT. Automatic non-rigid (Ablation-fit) or semi-automatic rigid (SAFIR) co-registration is performed to align the image volumes. Finally, MAM calculation is performed and visualized in two-dimensional and 3D views

Hereafter, 3D MAM quantification was automatically obtained in each software. For Ablation-fit, incomplete tumor coverage was labeled by the software as residual tumor, and was classified as MAM < 0 mm. For remaining ablations, unablated volumes for all 3D safety margins from 1 mm to 10 mm were calculated. The MAM was defined as the maximum 3D safety margin that was completely ablated. For SAFIR, the MAM was calculated as the smallest 3D distance between the tumor and ablation zone rounded to the nearest millimeter, where negative values indicate incomplete coverage. For both software, the side(s) of the tumor where suspected insufficient margins (< 5 mm) occurred were documented by dividing the tumor in octants along the left-right, cranio-caudal, and anterior-posterior axes. A schematic overview of MAM quantification is summarized in Fig. 2. Additional details are provided in S1.

### Statistical analyses

The primary endpoint was to determine whether the MAM was independently associated with LTP per software. Secondary endpoints were the diagnostic performance of MAM generated by each software to discriminate between cases with and without LTP and per-tumor inter-software agreement in MAM quantification. Uni- and multivariable analyses for LTP-free survival (LTPFS) were performed using the Cox proportional hazard regression model. Hazard ratios (HR) and 95% confidence intervals (CI) were calculated. Survival curves were estimated using the Kaplan–Meier method and differences were assessed using the log-rank test. ROC analysis was used to determine diagnostic performance and area under the curve (AUC) of MAM generated by each software. For cases with MAM < 0 mm or > 10 mm, Ablation-fit does not provide absolute MAM values precluding direct comparison between both software. For cases with MAM between 0 mm and 10 mm, inter-software agreement was assessed using single-measurement, absolute agreement intraclass correlation coefficient (ICC), and absolute difference. Continuous data are expressed as means ± standard deviation with range or medians with interquartile range (IQR) or range. The Shapiro-Wilk method was used for normality testing. A *p* value <  0.05 was considered statistically significant. All statistical analyses were performed using SPSS (v23.0; IBM) and R (v3.6.3; R Foundation).

## Results

### Patient cohort and baseline demographics

Overall, 113 patients who underwent thermal ablation for 189 CRLM met the inclusion criteria. A total of 173/189 (92%) CRLM could be successfully analyzed using both software and could be included for final analysis. Sixteen tumors were excluded based on suboptimal tumor visibility to allow segmentation (*n* = 4) or failed image co-registration (*n* = 12) (Fig. 1).

The final cohort thus consisted of 103 patients (mean age: 67 years ± 10 [SD]; 78 men) treated in 120 sessions (median number of treated CRLM per session: 1 [IQR: 1–2]) for 173 CRLM (mean diameter: 1.9 cm ± 1.1 [SD]). The distribution of included CRLM per institution was 85 (VUMC), 73 (MUI), and 15 (RUMC). Baseline patient, procedure, and tumor characteristics are summarized in Table 1.

**Table 1:** Baseline patient, procedure, and tumor demographics

Patient characteristics	(*N* = 103)
Age, (y)	67 ± 10 (range: 31–88)
Sex	
Male	78 (76)
Female	25 (24)
Prior CTx	
No	60 (58)
Yes	43 (42)
Extrahepatic disease	
No	85 (82)
Yes	18 (18)
RAS mutation status*	
Wild-type	36 (65)
Mutant	19 (35)

Procedure characteristics	(*N* = 120)
No. of treated tumors per procedure	
1	81 (68)
2	27 (22)
3	10 (8)
4	2 (2)
Anesthesia technique	
General anesthesia	72 (65)
Propofol sedation	48 (35)
Contrast-enhancement technique	
Intravenous	64 (53)
Hepatic arteriography	56 (47)
Ablation modality	
Microwave	57 (48)
Radiofrequency	63 (52)

Tumor characteristics	(*N* = 173)
Tumor size, (cm)	
Mean	1.9 ± 1.1 (range: 0.3–5.5)
< 1	28 (16)
1–2	69 (40)
2–3	43 (25)
3–4	21 (12)
4–5	8 (5)
> 5	4 (2)
Tumor location	
Supcapsular	63 (36)
Non-subcapsular	110 (64)
Proximity to vessel	
Perivascular	35 (20)
Non-perivascular	138 (80)

Data are mean ± SD, with a range in parentheses or numbers, with the percentage in parentheses

^*^ Data was available from 55 patients

### Clinical outcomes

The median follow-up was 31 months (IQR: 22–47). Technical success was achieved in all (173/173) lesions. The overall incidence of LTP was 21/173 (12.1%) after a mean time to progression of 14.7 months. The per-center incidences of LTP were 4/85 (5%), 10/73 (14%), and 7/15 (47%).

### MAM quantification

**Fig. 3:**
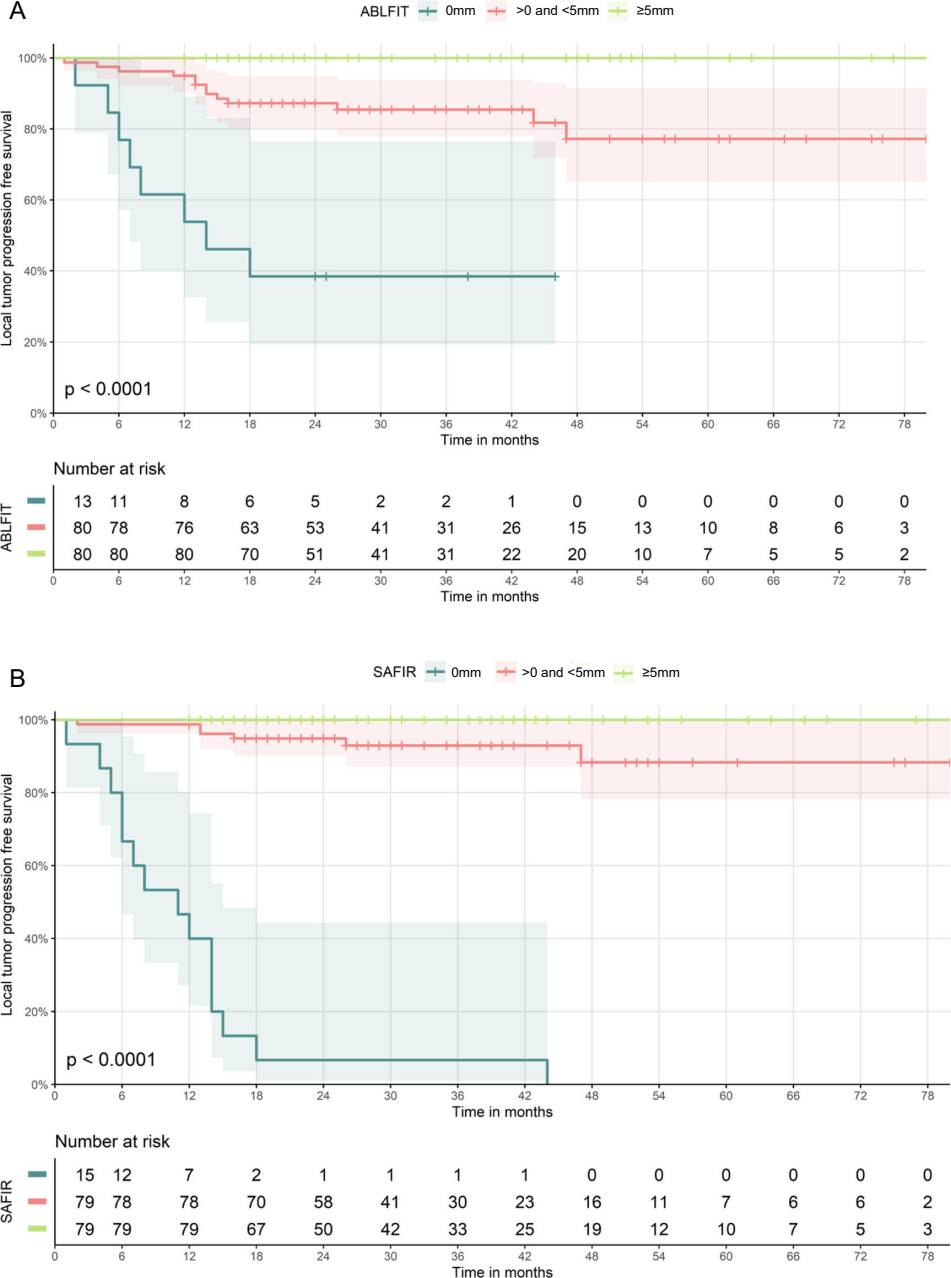
LTPFS curves stratified by MAM 0, > 0 and < 5, and > 5 mm for Ablation-fit (**A**) and SAFIR (**B**)

Of 173 ablated CRLM, MAM ≥ 5 mm; > 0 mm and < 5 mm; and ≤ 0 mm were measured in 80 (46.2%), 80 (46.2%), and 13 (7.6%) using Ablation-fit and 79 (45.7%), 79 (45.7%), and 15 (8.6%) using SAFIR. Of tumors with MAM ≥ 5 mm; > 0 mm and < 5 mm; and ≤ 0 mm measured using Ablation-fit, LTP was observed in 0/80 (0%), 13/80 (16.3%) and 8/13 (61.5%), respectively (Fig. 3a). Of tumors with MAM ≥ 5 mm; > 0 mm and < 5 mm; and ≤ 0 mm measured using SAFIR, LTP was observed in 0/79 (0%), 6/79 (7.6%) and 15/15 (100%), respectively (Fig. 3b). Smallest MAM where no LTP was observed was ≥ 4 mm for Ablation-fit and ≥ 5 mm for SAFIR. The side of LTP corresponded to the side(s) of the tumor treated with insufficient margins in 20/21 (95%) and 20/21 (95%) cases for Ablation-fit and SAFIR, respectively.

### LTPFS

Univariable Cox regression analysis showed that age (HR: 0.95, 95% CI: 0.92–0.99, *p* = 0.02), tumor size (HR: 1.04, 95% CI: 1.00–1.07, *p* = 0.045), and MAM of each software (Ablation-fit: HR: 0.51, 95% CI: 0.41–0.64, *p* < 0.001; SAFIR: HR: 0.49, 95% CI: 0.41–0.59, *p* < 0.001) were significantly associated with LTPFS (Table 2). On multivariable Cox regression analysis, the MAM of each software (Ablation-fit: HR: 0.47, 95% CI: 0.36–0.61, *p* < 0.001; SAFIR: HR: 0.42, 95% CI: 0.32–0.55, *p* < 0.001) were the only independent factors significantly associated with LTPFS (Table 3).

**Table 2:** Univariable analysis of factors associated with LTPFS

	HR, (95% CI)	*p* value
Age	0.95 (0.92–0.99)	0.02
Sex		
Male	Reference	
Female	1.10 (0.68–1.76)	0.70
Prior CTx		
No	Reference	
Yes	1.14 (0.48–2.68)	0.77
Tumor size	1.04 (1.00–1.07)	0.045
Tumor location		
Non-subcapsular	Reference	
Subcapsular	0.74 (0.29–1.91)	0.53
Proximity to vessel		
Non-perivascular	Reference	
Perivascular	0.91 (0.31–2.70)	0.86
Ablation type		
MWA	Reference	
RFA	0.94 (0.39–2.23)	0.88
MAM		
Ablation-fit	0.51 (0.41–0.64)	< 0.001
SAFIR	0.49 (0.41–0.59)	< 0.001

*CTx* chemotherapy, *MWA* microwave ablation, *RFA* radiofrequency ablation, *HR* hazard ratio, *CI* confidence interval, *MAM* minimal ablative margin

**Table 3:** Multivariable analysis of factors associated with LTPFS

	Ablation-fit^†^		SAFIR^†^	
	HR, (95% CI)	*p* value	HR, (95% CI)	*p* value
Age	0.97 (0.93–1.00)	0.08	1.05 (0.99–1.10)	0.10
Sex				
Male	Reference		Reference	
Female	2.80 (0.97–8.12)	0.06	0.48 (0.13–1.79)	0.28
Prior CTx				
No	Reference		Reference	
Yes	1.86 (0.61–5.68)	0.28	1.85 (0.59–5.85)	0.29
Tumor size	1.02 (0.98–1.07)	0.31	0.99 (0.95–1.04)	0.81
Tumor location				
Non-subcapsular	Reference		Reference	
Subcapsular	0.54 (0.18–1.63)	0.27	0.35 (0.09–1.36)	0.13
Proximity to vessel				
Non-perivascular	Reference		Reference	
Perivascular	0.79 (0.23–2.75)	0.71	0.27 (0.05–1.40)	0.12
Ablation type				
MWA	Reference		Reference	
RFA	1.78 (0.54–5.95)	0.35	1.82 (0.50–6.55)	0.36
MAM	0.47 (0.36–0.61)	< 0.001	0.42 (0.32–0.55)	< 0.001

*CTx* chemotherapy, *MWA* microwave ablation, *RFA* radiofrequency ablation, *HR* hazard ratio, *CI* confidence interval, *MAM* minimal ablative margin

^†^A separate multivariable model was fitted with the MAM of each software

### Diagnostic performance

**Fig. 4:**
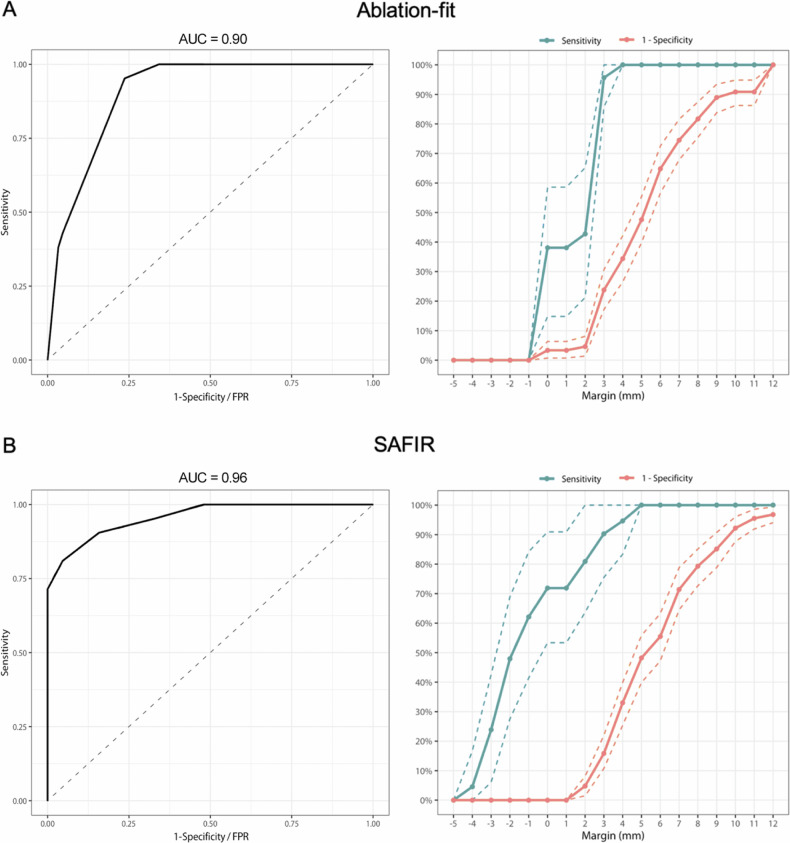
ROC-curves (left) and LTP detection rate (sensitivity) and false positive rate (1-specificity) for different MAM thresholds (right) for MAM measured by Ablation-fit (**A**) and SAFIR (**B**). Dashed lines indicate 95% CI

ROC analysis showed excellent diagnostic performance for discriminating between cases with and without LTP of MAM measured by Ablation-fit (AUC: 0.90, 95% CI: 0.86–0.95) and SAFIR (AUC: 0.96, 95% CI: 0.91–1.00). The detection rate of LTP (sensitivity) and false positive rate (1-specificity) as a function of MAM thresholds are shown for each software (Fig. 4). Corresponding sensitivity and false positive rate values for MAM thresholds between 0 mm and 5 mm are shown in Table 4.

**Table 4:** Sensitivity and false positive rate per software for MAM thresholds between 0 mm and 5 mm

MAM threshold^*^, (mm)	Ablation-fit		SAFIR	
	Sensitivity	FPR	Sensitivity	FPR
0	0.38	0.03	0.71	0.00
1	0.38	0.03	0.71	0.00
2	0.43	0.05	0.81	0.05
3	0.95	0.24	0.91	0.16
4	1.00	0.34	0.95	0.33
5	1.00	0.48	1.00	0.48

*MAM* minimal ablative margin, *FPR* false positive rate

^*^ Positive test is considered when MAM is less than the cut-off point

### Inter-software agreement

Inter-software agreement could be calculated for 147/173 tumors with a MAM between 0 mm and 10 mm. The per-tumor ICC showed moderate agreement for MAM quantification between both software and was 0.53 (95% CI: 0.41–0.64). The per-tumor median difference in MAM quantification between both software was 1 mm (IQR: −2 mm to 2 mm) and the median absolute difference was 2 mm (IQR: 1–3 mm).

## Discussion

Achieving sufficient ablative margins has been shown critical to improve local control following thermal ablation of CRLM in multiple single-center studies evaluating single software approaches. In this study, we found the 3D MAM derived from intraprocedural contrast-enhanced pre- and post-ablation CT imaging to be the only independent predictor of LTP following thermal ablation of CRLM across multicenter data and two dedicated ablation confirmation software. Ablations achieving a MAM ≥ 4 mm for Ablation-fit and ≥ 5 mm for SAFIR were associated with local control. Consistency of these findings across two different software, different users, and intraprocedural CT data from three institutions represents an important step toward clinical integration.

Current literature investigating the relation between ablative margins and LTP is characterized by a wide variety of margin quantitation methods, utilizing various combinations of preceding diagnostic, intraprocedural or follow-up imaging as the pre- and post-ablation references, as well as different imaging modalities [20]. Recent work has shown ablative margins quantified on intraprocedural CT to significantly outperform initial follow-up CT in predicting LTP following thermal ablation of CRLM [25]. This work utilized solely intraprocedurally acquired pre- and post-ablation CE-CT imaging, allowing the methodology to be applicable during the actual intervention itself. In addition to superior accuracy in predicting LTP, a fully intraprocedural method for ablation endpoint assessment is particularly desirable as to enable the ability to immediately re-ablate site(s) of inadequate coverage and potentially improve local control rates without the need for a re-do procedure. Importantly, identified site(s) at risk for LTP were co-localized with the site of actual LTP in > 95% of cases and were consistent between both software.

Several studies have investigated optimal cut-off points for margin adequacy in thermal ablation of CRLM. Initial studies using landmark-based visual comparison of diagnostic and initial follow-up CT imaging recommended a MAM greater than 5 mm, and preferably greater than 10 mm all around the tumor to achieve optimal local control [9, 11, 26, 27]. Recent studies that used 3D approaches to quantify the MAM utilizing rigid or non-rigid image co-registration techniques have indicated an ablative margin greater than 2–5 mm to be associated with no LTP [8, 16, 18]. In a large single-institutional cohort Lin et al recently demonstrated a MAM threshold greater than 5 mm as the optimal endpoint following CT-guided CRLM ablation [8]. Our study confirms these findings, where no LTP was observed for respective MAMs of ≥ 4 mm and ≥ 5 mm using both elastic deformation and rigid image fusion software. Nevertheless, with Ablation-fit and SAFIR we observed a MAM of ≥ 5 mm in only 46.2% and 45.7% of ablated tumors, respectively. This is particularly interesting given that in all cases the achievement of technical success including a ≥ 5 mm ablative margin was perceived by the treating physician. On the other hand, as shown in Table 4 this finding also implies that applying a ≥ 5 mm threshold as the ablation endpoint using the software evaluated in this work would yield a substantial false positive rate (~ 48%), i.e., lesions treated with margins < 5 mm which would not develop LTP. We additionally provided diagnostic performance as a function of different margin thresholds, which should be important data for determining the clinical implications of each margin endpoint. Given the different performances per software found in this study, the definition of optimal ablative margin endpoints should require careful validation for each ablation confirmation approach in order to optimize the identification of tumors at high risk of developing LTP while minimizing the number of false positives.

Accurate image co-registration of pre- and post-ablation imaging is a prerequisite for robust MAM quantification [28]. In this study, only a small subset (12/189; 6%) of initially included CRLM had to be excluded due to insufficient image registration quality in one or both software. Interestingly, the use of two different approaches (i.e., semi-automatic rigid vs. automatic non-rigid) for image registration between both software did not result in large differences in diagnostic performance. Possibly, this is due to the use of solely intraprocedural pre- and post-ablation imaging inherently reducing the between-scan variability as compared to preceding or follow-up imaging acquired at different time points. Nevertheless, our study found an average per-tumor margin discrepancy of 2 mm between both software, which indicates the confidence with which margins can be quantified in individual cases.

Finally, comparative trials and prospective evidence are needed to steer and confirm the impact of margin confirmation software on improving local control following CRLM ablation. The generation of an open, standardized multicenter dataset may be beneficial to serve as a benchmark for confirmation software performance evaluations and facilitate the determination of optimal margin endpoints before clinical integration. Furthermore, prospective trials, such as the ongoing ACCLAIM trial (NCT05265169), CIEMAR registry [29] and recently published COVER-ALL study protocol [30] are urgently needed to support clinical adoption of margin confirmation to confirm treatment adequacy. Particularly considering the evolving role of thermal ablation in the management of CRLM, currently studied as an equivalent to surgical resection in phase III randomized controlled COLLISION trial [31], optimization of treatment outcomes and standardization of outcome evaluation appear desirable.

Our study had several limitations. First, our study pertains to a retrospective non-consecutive multicenter cohort which was selected based on the inclusion criteria. Although these were mostly related to the exclusion of cases with prior local treatment of the target tumor and insufficient follow-up, this may have introduced a bias to our results. Yet, this study presents the first large multicenter cohort evaluating MAM quantitation after CRLM ablation utilizing solely intraprocedural CT imaging. Furthermore, due to inherent differences between both ablation confirmation software, the MAM could not be calculated in the ranges < 0 mm and > 10 mm in one software, precluding the direct comparison of MAM results for these cases. Also, this study did not evaluate whether immediate re-ablation within the same session (or second-hit) could have been performed in each case of LTP, therefore restricting its clinical implication. Finally, even though a two-reader consensus approach was applied the semi-automatic assessments used in the study are subject to reader variability, which requires further study.

In conclusion, MAM was independently associated with LTP after thermal ablation of CRLM across multicenter data and two confirmation software. Ablations achieving a MAM ≥ 5 mm were associated with local control in both software. Ultimately, optimal cut-off points for margin adequacy and its implications for clinical use require validation per confirmation software.

## Supplementary information

ELECTRONIC SUPPLEMENTARY MATERIAL

## Abbreviations

AUC: Area under the curve
CE-CT: Contrast-enhanced CT
CRLM: Colorectal liver metastasis
3D: Three-dimensional
HR: Hazard ratio
ICC: Intraclass correlation coefficient
LTP: Local tumor progression
MAM: Minimal ablative margin
