# Supplementary material for: Multicenter and inter-software evaluation of ablative margins after thermal ablation of colorectal liver metastases
Source: Eur Radiol. 2024 Aug 2;35(2):1046–56. doi: 10.1007/s00330-024-10956-5 (PMC11782453; doi:10.1007/s00330-024-10956-5)
Supplement: Supplementary file 1 — ELECTRONIC SUPPLEMENTARY MATERIAL [file 330_2024_10956_MOESM1_ESM.pdf]

Multicenter and Intersoftware Evaluation of Ablative Margins after Thermal Ablation of Colorectal Liver Metastases

ELECTRONIC SUPPLEMENTARY MATERIAL

Appendix - Supplementary 1

Materials & Methods

Definitions

Tumors were classified as perivascular when located within 5 mm from a major (>4 mm) hepatic vessel based on the pre-ablation CE-CT scan. Subcapsular location was defined as tumor located within 5 mm from the liver edge.

Tumor segmentation was preferably performed in the portal-venous phase scans, but in cases where the tumor was deemed better delineable in the arterial phase, the latter could be used. Tumor segmentation followed the boundary separating the hypodense region from adjacent liver parenchyma (portal-venous phase) or circumferential arterial rim enhancement (arterial phase). Ablation zone segmentation was performed in the portal-venous phase scans and adhered to the non-perfused area compared to surrounding liver parenchyma.

For subcapsular located lesions, all voxels outside of the liver parenchyma on the post-ablation CE-CT were considered ablated and MAM quantification was restricted to non-ablated liver parenchyma.

Statistical analysis

AUC values were interpreted as follows: <0.70 low performance, 0.70–0.80 moderate performance, 0.80-0.90 high performance and 0.90-1.00 excellent performance. ICC values were interpreted as follows: 0-0.50 poor agreement, 0.50-0.75 moderate agreement, 0.75-0.90 good agreement and 0.90-1.00 excellent agreement.

Power calculation

Overall incidence of local recurrence is estimated at 15%. Based on a previous single-center study using Ablation-fit (1) and a recent study by Lin et al. (2), we assumed 3-year cumulative incidence rate of local recurrence of 25% for ablations achieving a MAM <5 mm and 5% for a MAM ≥5 mm with the proportion of ablations with MAM ≥5 mm being 46% (allocation ratio of 1 : 0.85). At an overall sample size of 189 tumors, accounting for 10% exclusion rate, a two-sided log-rank test has 80% power at the 0.05 significance level to detect a hazard ratio of 0.37.

Results

Influence of RAS Mutation Status, Anesthesia Technique, Contrast Enhancement Technique, Proximity to Vessel and Tumor Size on MAM

The Mann-Whitney *U* Test was used to assess the effect of RAS mutation status, anesthesia technique, contrast administration technique, proximity to vessel and tumor size on the MAM. Spearman's rho was used to assess correlation between tumor size and MAM.

For RAS mutation status, data was available for 55/103 patients and 88/173 tumors. For Ablation-fit, the median MAM was 5 mm (IQR, 2-7) for RAS wild-type tumors (60/88) and 4 mm (IQR, 2-6) for RAS mutant tumors (28/88) without statistical significance (*P*=.38). For SAFIR, the median MAM was 4 mm (IQR, 2-7) for RAS wild-type tumors and 4.5 mm (IQR, 4-6) for RAS mutant tumors without statistical significance (*P*=.35).

For anesthesia technique, the median MAM using Ablation-fit was 4 mm (IQR, 2-6) for tumors treated under propofol sedation (74/173) and 4 mm (IQR, 2-7) for tumors treated under general anesthesia (99/173) without statistical significance (*P*=.55). For SAFIR, the median MAM was 4 mm (IQR, 3-6) for propofol sedation and 4 mm (IQR, 2-7) for general anesthesia without statistical significance (*P*=.31).

For contrast enhancement technique, the median MAM using Ablation-fit was 4 mm (IQR, 2-6) when intravenous contrast injection was used (87/173) and 4 mm (IQR, 2-6) when catheter-based hepatic arteriography was used (86/173) (*P*=.64). For SAFIR, median MAM was 4 mm (IQR, 2-7) for intravenous injection and 4 mm (IQR, 3-6) for hepatic arteriography (*P*=.82).

For proximity to vessel, the median MAM using Ablation-fit was 4 mm (IQR, 2-6) for perivascular tumors (35/173) and 4 mm (IQR, 2-6) for non-perivascular tumors (138/173) (*P*=.64). For SAFIR, the median MAM was 3 mm (IQR, 2-5) for perivascular tumors and 5 mm (IQR, 3-7) for non-perivascular tumors, with statistical significance (*P*=.02).

For tumor size, the median MAM was 5 mm (IQR, 2-7) for tumors ≤2 cm (110/173) vs. 4 mm (IQR, 2-5) for tumors >2 cm (63/173) (*P*=.12) using Ablation-fit and 4.5 mm (IQR, 3-7) vs. 4 mm (IQR, 2-7) (*P*=.11) using SAFIR. Spearman correlation coefficient between tumor size and MAM was -0.1 (*P*=.44) for Ablation-fit and -0.1 (*P*=.29) for SAFIR.

References

1. Laimer G, Jaschke N, Schullian P, Putzer D, Eberle G, Solbiati M, Solbiati L, Goldberg SN, Bale R. Volumetric assessment of the periablational safety margin after thermal ablation of colorectal liver metastases. Eur Radiol. 2021 Sep;31(9):6489-6499.
2. Lin YM, Paolucci I, O'Connor CS, Anderson BM, Rigaud B, Fellman BM, Jones KA, Brock KK, Odisio BC. Ablative Margins of Colorectal Liver Metastases Using Deformable CT Image Registration and Autosegmentation. Radiology. 2023 Apr;307(2):e221373.
